# Supplementary material for: Galectin-3 deficiency drives lupus-like disease by promoting spontaneous germinal centers formation via IFN-γ
Source: Nat Commun. 2018 Apr 24;9:1628. doi: 10.1038/s41467-018-04063-5 (PMC5915532; doi:10.1038/s41467-018-04063-5)
Supplement: Supplementary file 1 — Supplementary Information [file 41467_2018_4063_MOESM1_ESM.pdf]

Supplementary Information

Galectin-3 deficiency drives lupus-like disease by promoting spontaneous germinal centers formation via IFN-γ

Beccaria et al.

Supplementary Figures

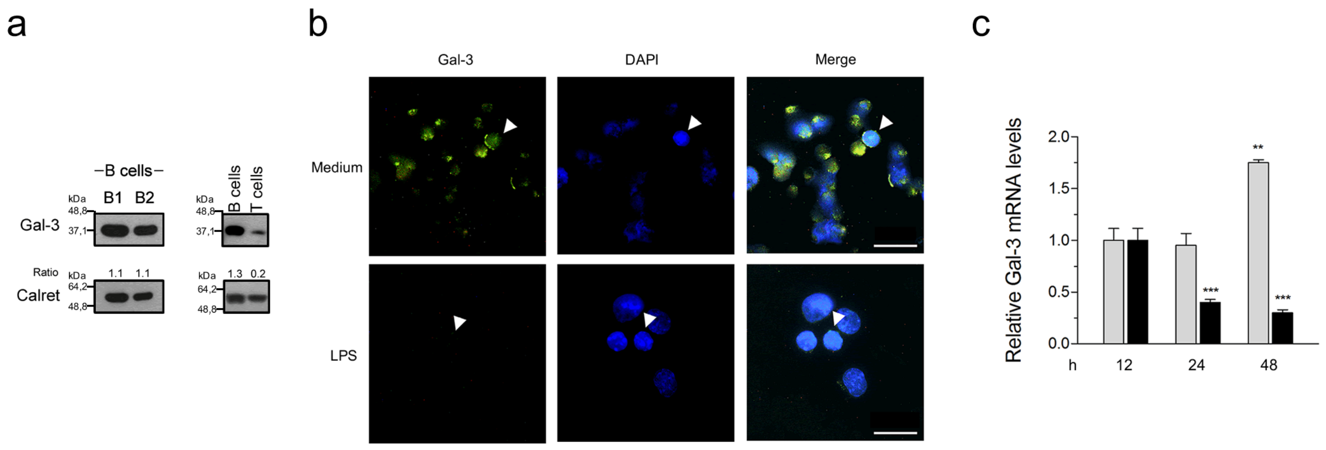

Supplementary Figure 1. Down-regulation of Gal-3 during PC differentiation.
(**a**) Comparative western blot of the expression of Gal-3 in murine ex-vivo peritoneal B1 cell (B1) and follicular splenic B cell (B2), and total splenic B and T cells. Gal-3 signal was normalized to the loading control calreticulin (Calret), and ratios are shown between the pictures (n = 5 for each condition). (**b**) Immunofluorescence for Gal-3 expression in splenic B cells cultured with Medium (upper panels) or LPS (lower panels) for 72 h. The specific Gal-3 signal is shown in green. Nuclei were counter-stained with DAPI (blue). White arrowheads indicate the presence (upper panels) or absence of Gal-3 (lower panels) in B cells cultures with medium and LPS, respectively (n = 4 for each condition). Scale bar, 20 µm (**c**) Quantitative RT–PCR analysis of Gal-3 mRNA levels of purified splenic B cells cultured with Medium (grey bars) or LPS (black bars) for 12, 24 and 48 h (n = 6 for each condition). ** $p < 0.01$; *** $p < 0.001$ (one-way ANOVA; Tukey–Kramer multiple comparison test). Data are representative of three independent experiments.

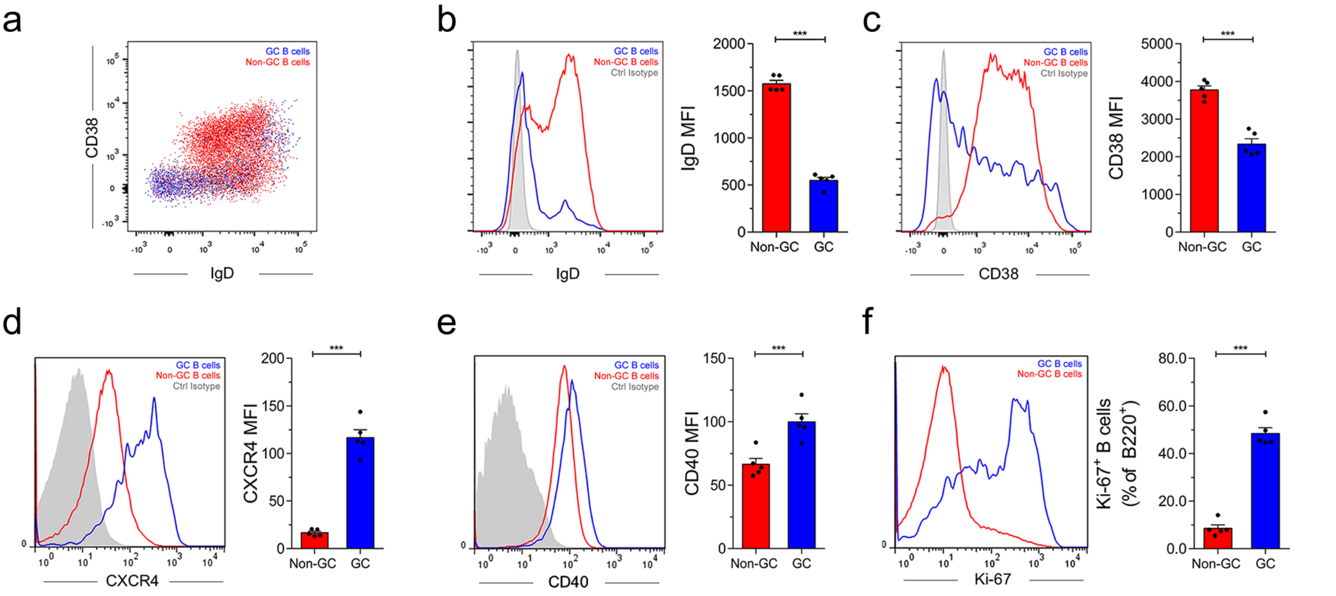

Supplementary Figure 2. Deep characterization of GC B cells in Gal-3 KO mice.
(**a**) Representative dot plot of CD38 vs IgD of gated B220$^+$ cells from the spleen of Gal-3 KO mice showing GC (blue) and non-GC B cells (red) (*n* = 5 for both strains). (**b-e**) Representative histograms and statistical analysis of mean fluorescence intensity (MFI) of IgD (**b**), CD38 (**c**), CXCR4 (**d**) and CD40 (**e**) on GC (B220$^+$ CD4$^-$ GL7$^+$ FAS$^+$) and non-GC (B220$^+$ CD4$^-$ GL7$^-$ FAS$^-$) B cells from 8 week-old Gal-3 KO mice (*n* = 5 for both strains). (**f**) Representative histogram and statistical analysis of the frequency of Ki-67$^+$ cells in GC and non-GC B cells from 8 week-old Gal-3 KO mice (*n* = 5 for both strains). *** $p < 0.001$ (paired Student´s t-test). Data are representative of three independent experiments.

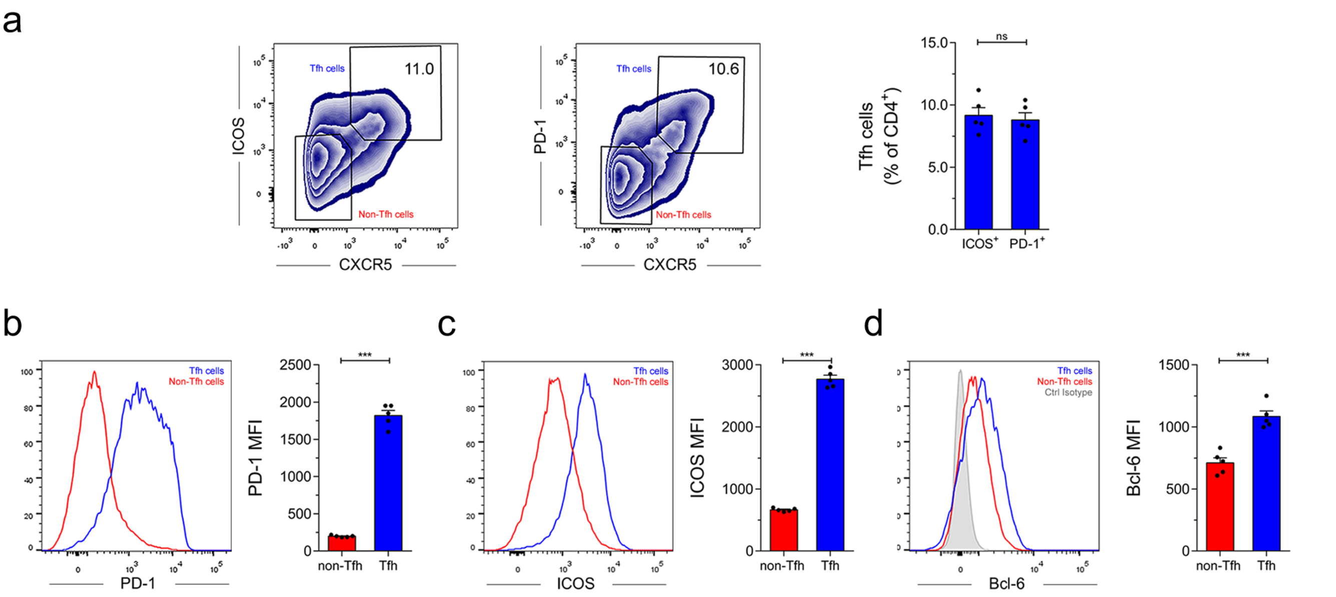

Supplementary Figure 3. Identification of bonafide Tfh cells in Gal-3 KO mice.
(**a**) Representative dot plot and statistical analysis of the frequency of Tfh cell from spleen of 8-week-old Gal-3 KO mice identified by expression of $CXCR5^+$ $ICOS^+$ (left) or $CXCR5^+$ $PD\text{-}1^+$ (right) gated on $CD4^+$ $Foxp3^-$ T cells (n = 5 for both strains). (**b**) Representative histograms and statistical analysis of Mean Fluorescence Intensity (MFI) of PD-1 staining in Tfh cell ($CD4^+$ $CXCR5^+$ $ICOS^+$ $Foxp3^-$) and non-Tfh ($CD4^+$ $CXCR5^-$ $ICOS^-$ $Foxp3^-$) from spleen of 8-week-old Gal-3 KO mice (n = 5 for both strains). (**c**) Representative histograms and statistical analysis of MFI of ICOS staining on Tfh cell ($CD4^+$ $CXCR5^+$ $PD\text{-}1^+$ $Foxp3^-$) and non-Tfh ($CD4^+$ $CXCR5^-$ $PD\text{-}1^-$ $Foxp3^-$) from spleen of 8-week-old Gal-3 KO mice (n = 5 for both strains). (**d**) Representative histograms and statistical analysis of MFI of Bcl-6 staining in Tfh cell ($CD4^+$ $CXCR5^+$ $ICOS^+$ $Foxp3^-$) and non-Tfh ($CD4^+$ $CXCR5^-$ $ICOS^-$ $Foxp3^-$) from spleen of 8-week-old Gal-3 KO mice (n = 5 for both strains). *** $p < 0.001$ (paired Student's t-test); ns, not significant. Data are representative of three independent experiments.

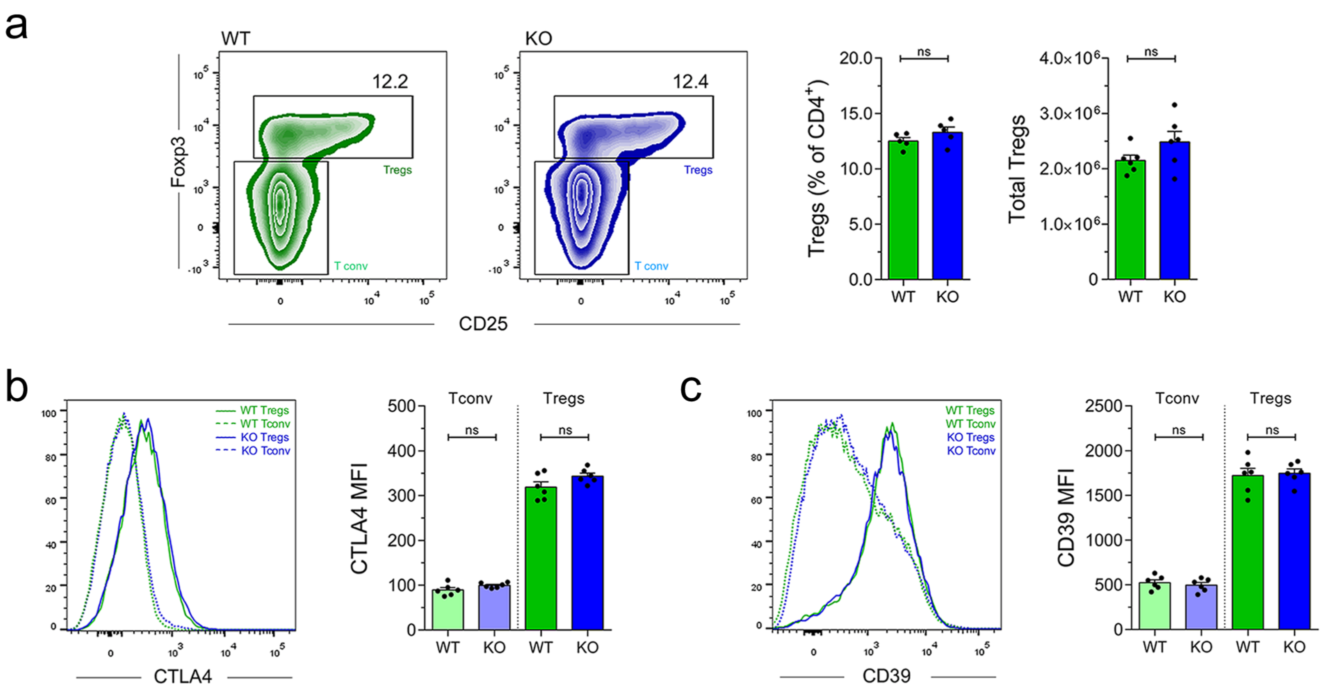

Supplementary Figure 4. Comparative study of regulatory T cells.
(**a**) Representative dot plot and statistical analysis of the frequency and number of Tregs cells (CD4$^+$ Foxp3$^+$ CD25$^+$) (*n* = 6 for both strains). (**b-c**) Representative histogram and statistical analysis of the Mean Fluorescence Intensity (MFI) of CTLA4 (**b**) and CD39 (**c**) on Tregs cell (CD4$^+$ Foxp3$^+$ CD25$^+$; dark colors) and conventional CD4$^+$ T cells (CD4$^+$ Foxp3$^-$ CD25$^-$; light colors) from spleen of 8-week-old WT (green) and Gal-3 KO mice (blue) (*n* = 6 for both strains). ns, not significant (unpaired Student´s t-test). Data are representative of three independent experiments.

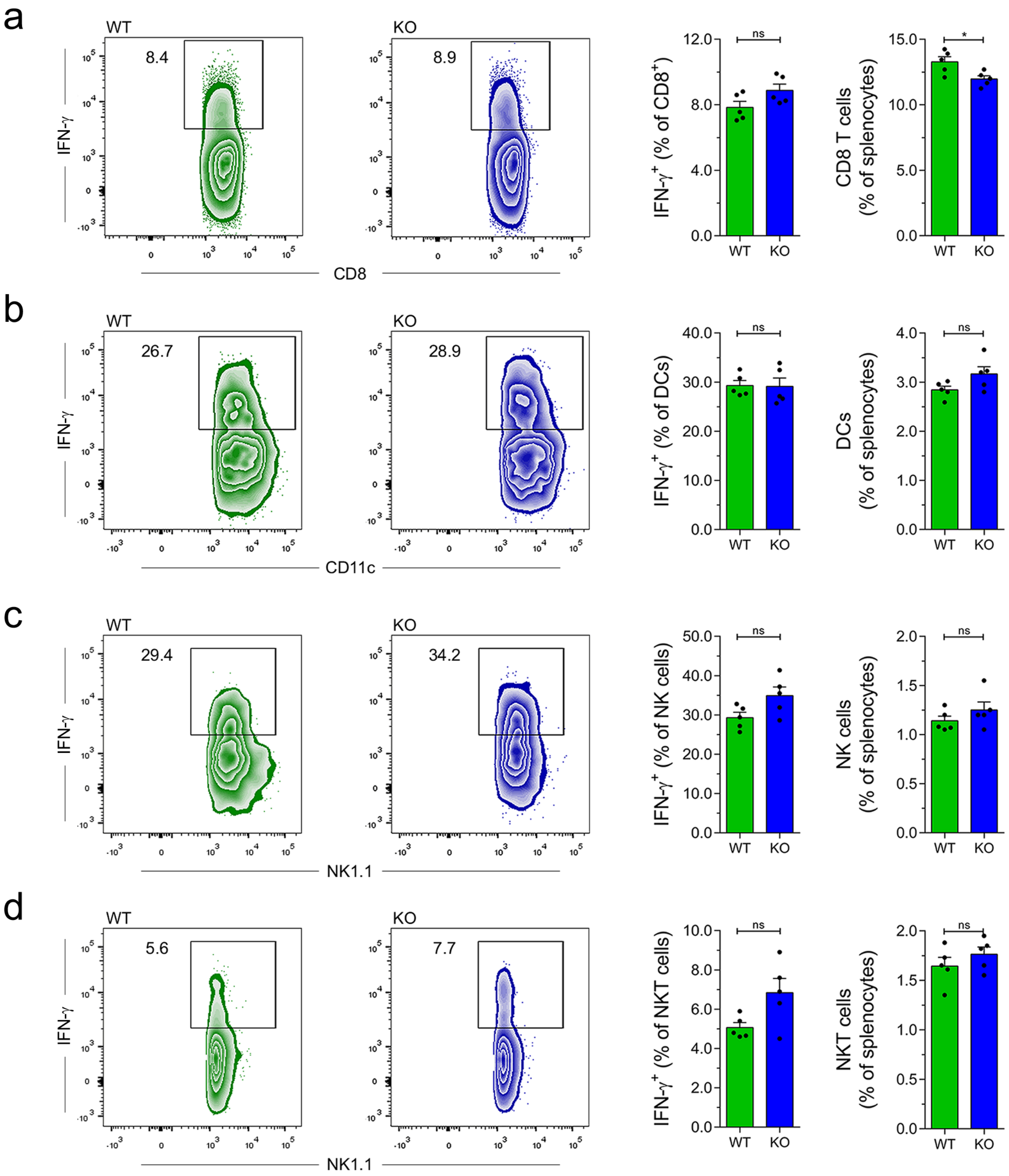

Supplementary Figure 5. IFN-γ-producing non-CD4+ T cells.
(**a-d**) Representative plots and statistical analysis of the frequency of IFN-γ-producing $CD8^+$ T cells ($CD3^+$ $CD8^+$ $CD4^-$) (**a**), DC cells ($CD11c^+$ IA/IE^+ $CD3^-$ $CD19^-$) (**b**), NK cells ($NK1.1^+$ $CD49b^+$ $CD3^-$ $TCR\beta^-$) (**c**) and NKT cells ($NK1.1^+$ $CD49b^+$ $CD3^+$ $TCR\beta^+$) (n = 5 for both strains) (**d**). Bar graphs on the right show the percentage of each indicated population in the spleen. * p <0.05; ns, not significant (unpaired Student's t-test). Data are representative of three independent experiments.

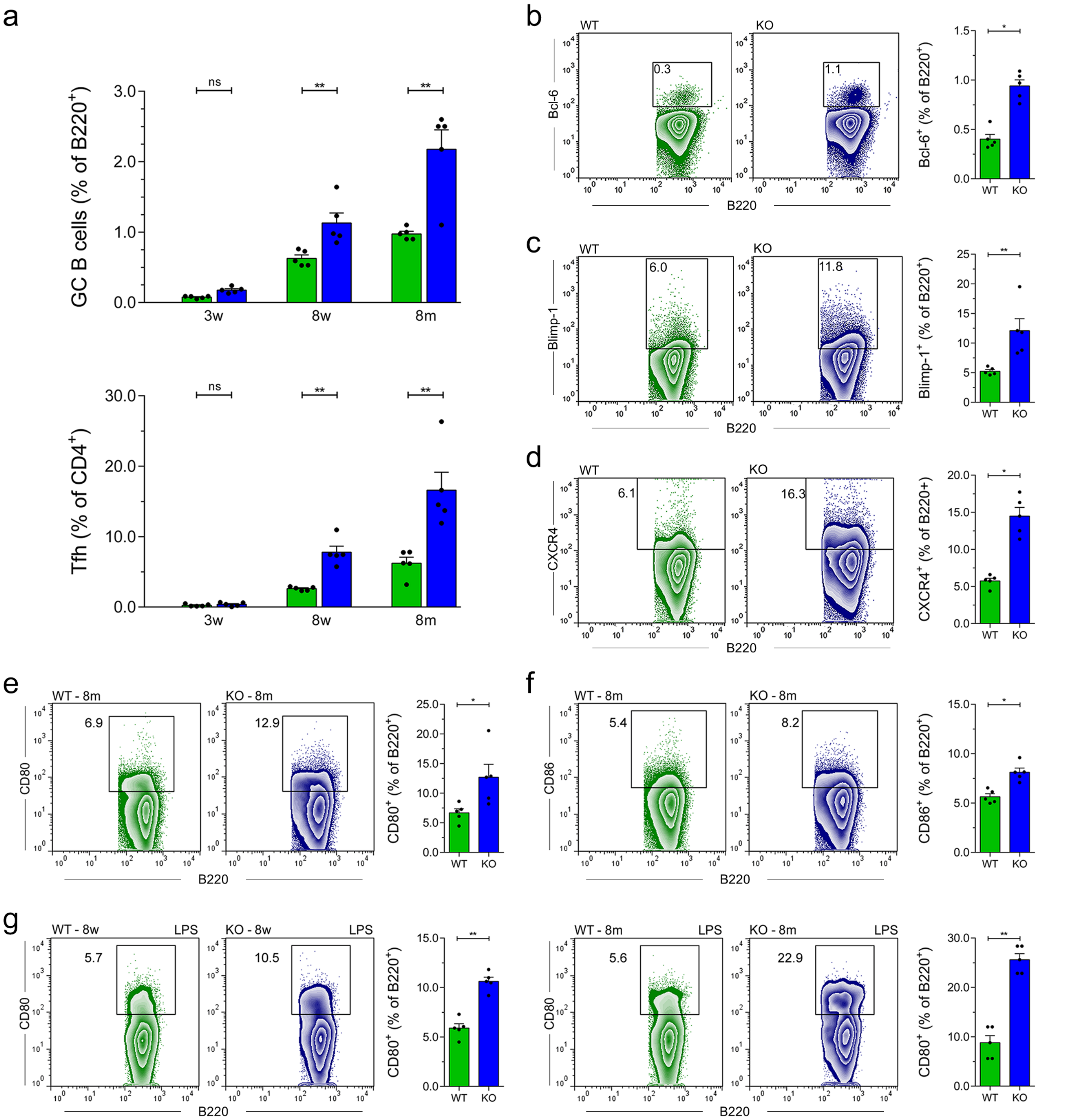

Supplementary Figure 6. Aged Gal-3 KO mice exhibit an activated B cell phenotype
(**a**) Bar graphs of the frequency of GC B cells (upper graph) and Tfh (lower graph) in the spleen from 3-week-, 8-week- or 8-month-old WT and Gal-3 KO mice (*n* = 5 per group at each time point). (**b-d**) Representative plots and statistical analysis of the frequency of Bcl-6+, CXCR4+ and Blimp-1+ B220+ B cells in the spleen from 8-month-old WT and Gal-3 KO mice, respectively (*n* = 5 for both strains). (**e-f**) Representative plots and statistical analysis of the percentages of CD80+ and CD86+ B cells of the spleen from 8-month-old WT (green) and Gal-3 KO (blue) mice (*n* = 5 for both strains). (**g**) Representative plots and statistical analysis of the percentages of CD80+ B cells of the spleen from 8-week (left) and 8-month-old (right) WT (green) and Gal-3 KO (blue) mice cultured with LPS for 24h (*n* = 5 for each condition). * $p < 0.05$, ** $p < 0.005$ (unpaired Student's t-test). Data are representative of three independent experiments.

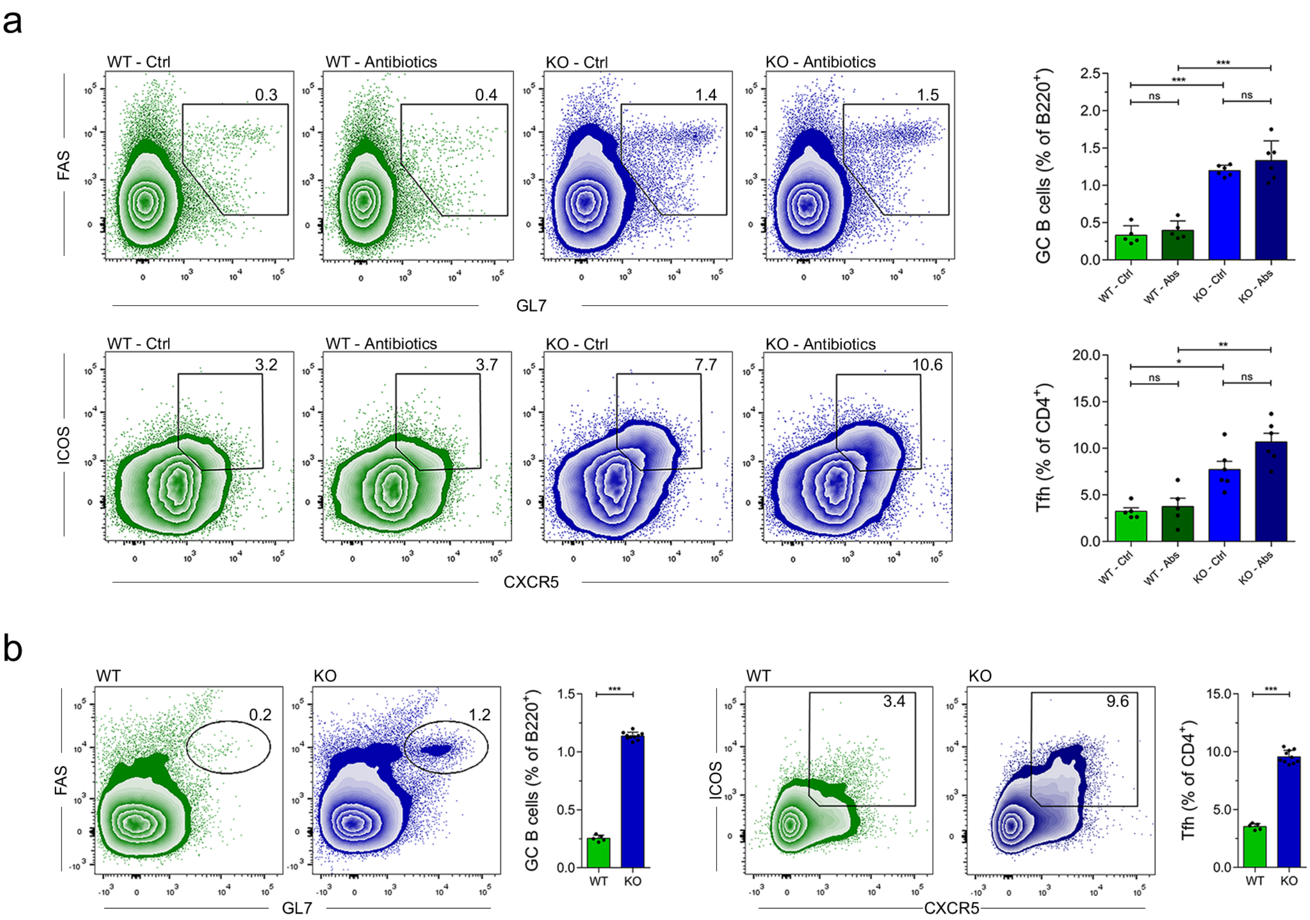

Supplementary Figure 7. Antibiotic treatment and co-housed experiments does not modify the frequency of GC B cells nor Tfh cells in Gal-3 KO mice.

(**a**) Representative plots and statistical analysis of the frequency of GC B cells ($B220^+$ FAS^+ $GL7^+$ $CD3^-$; upper panels) and Tfh cells ($CD4^+$ $CXCR5^+$ $ICOS^+$ $Foxp3^-$ $B220^-$; lower panels) in spleen from WT (green) and Gal-3 KO (blue) mice receiving water (controls, indicated as Ctrl) or antibiotics (indicated as Antibiotics) (WT n = 5; Gal-3 KO n = 6). (**b**) Representative plots and statistical analysis of the frequency of GC B cells (left panels) and Tfh cells (right panels) in the spleen of co-housed WT (green) and Gal-3 KO (blue) (WT n = 5; Gal-3 KO n = 10). * $p < 0.05$, ** $p < 0.005$, *** $p < 0.001$. ns, not significant (one-way ANOVA; Tukey–Kramer multiple comparison test was used for experiments displayed in a and unpaired Student's t-test was used in b). Data are representative of two independent experiments.

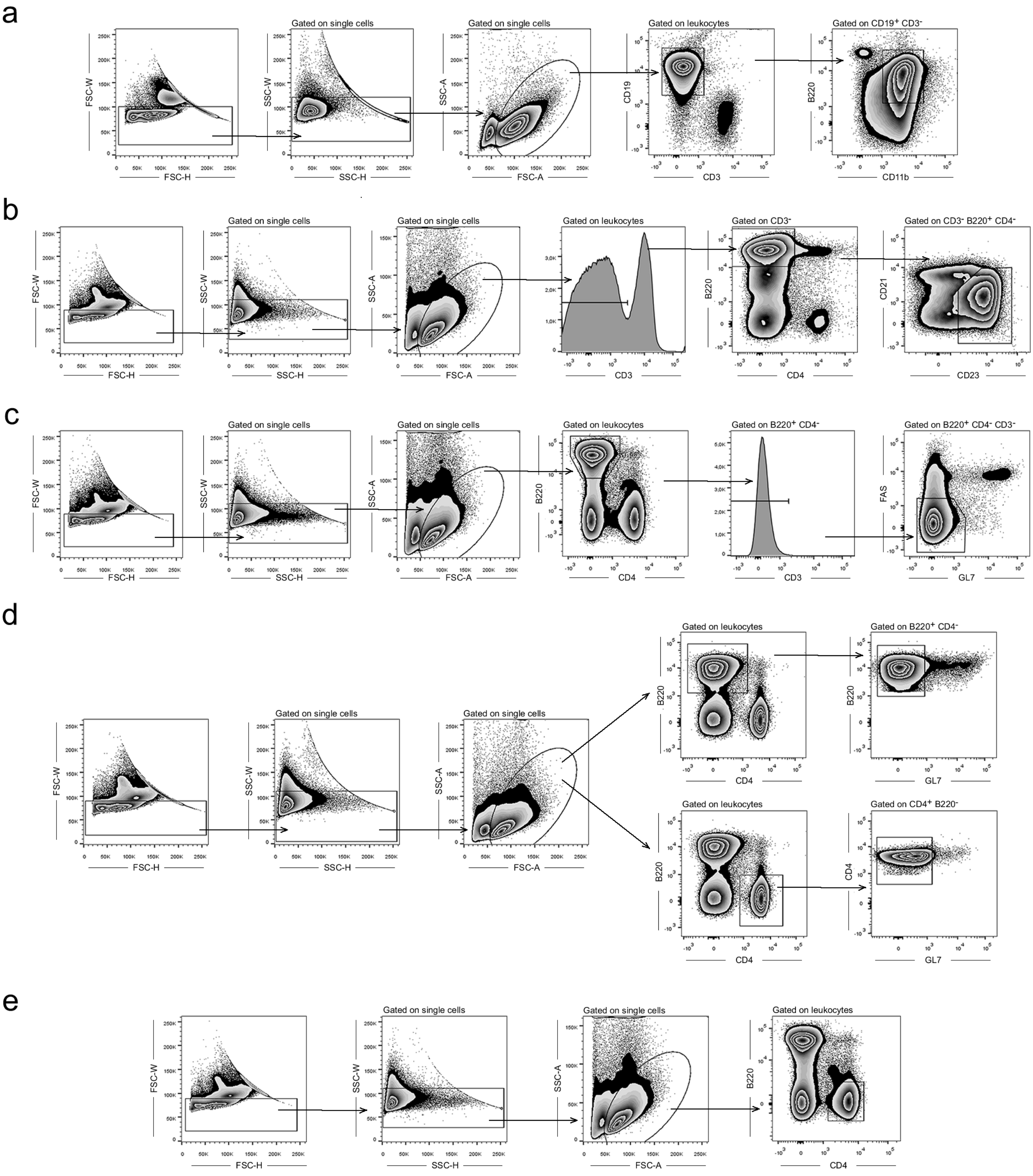

Supplementary Figure 8. Gating strategies used for cell sorting.
(**a**) Gating strategy to sort peritoneal B1 cells (CD19^{+} B220^{+} CD11b^{+} CD3^{-}) from WT mice for experiments presented on Supplementary Figure 1a. (**b**) Gating strategy to sort follicular splenic B2 cells (B220^{+} CD21^{+} CD23^{high} CD3^{-} CD4^{-}) from WT mice for experiments presented on Supplementary Figure 1a. (**c**) Gating strategy to sort non-GC B cells (B220^{+} FAS^{-} GL7^{-} CD3^{-} CD4^{-}) from WT and Gal-3 KO mice for experiments presented on Fig. 3b. (**d**) Gating strategy to sort B cells (B220^{+} GL7^{-} CD4^{-}) and CD4+ T cells (CD4^{+} GL7^{-} B220^{-}) from WT and Gal-3 KO mice for experiments presented on Fig. 3c. (**e**) Gating strategy to sort CD4^{+} T cells (CD4^{+} B220^{-}) from WT and Gal-3 KO mice for experiments presented on Fig. 4a.

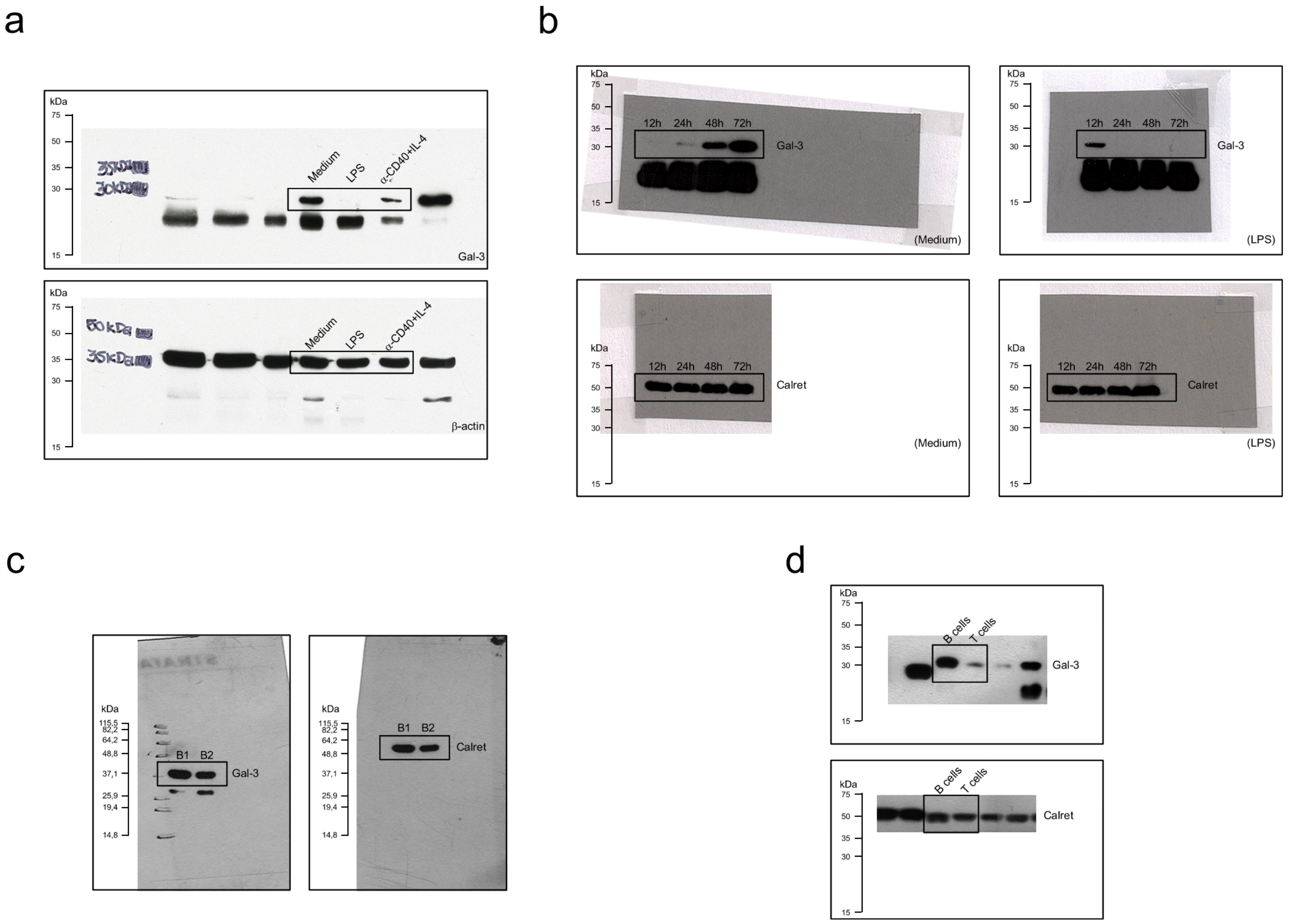

Supplementary Figure 9. Original data of immunoblots.
(**a**) Immunoblot data corresponding to Fig. 1a. (**b**) Immunoblot data corresponding to Fig. 1b. (**c**) Immunoblot data corresponding to Supplementary Figure 1a (left immunoblot). (**d**) Immunoblot data corresponding to Supplementary Figure 1a (right immunoblot).

Supplementary Table

Specificity and Fluorochrome	Clone	Catalog Number	Source	Dilution
Anti-B220 FITC	RA3-6B2	103206	BioLegend	1/200
Anti-B220 PE	RA3-6B2	103208	BioLegend	1/400
Anti-B220 PE-Cy7	RA3-6B2	103222	BioLegend	1/400
Anti-CD3ε PerCP-Cy5.5	145-2C11	100328	BioLegend	1/200
Anti-CD86 APC	GL-1	105012	BioLegend	1/200
Anti-CD4 APC-Cy7	GK1.5	100414	BioLegend	1/200
Anti-CD23 FITC	B3B4	101606	BioLegend	1/100
Anti-IFN-γ FITC	XMG1.2	505806	BioLegend	1/100
Anti-Bcl-6 PE	7D1	358504	BioLegend	1/75
Anti-IL-21R APC	4A9	131910	BioLegend	1/100
Anti-ICOS PE-Cy7	C398.4A	313520	BioLegend	1/100
Anti-IgD Alexa Fluor 594	11-26c.2a	405740	BioLegend	1/200
Anti-IFN-γRα Biotin	2E2	112804	BioLegend	1/200
Anti-NK1.1 PE-Cy7	PK136	108714	BioLegend	1/200
Anti-TCR β chain APC	H57-597	109212	BioLegend	1/200
Anti-B220 APC	RA3-6B2	17-0452-83	eBioscience	1/400
Anti-CD11b FITC	M1/70	11-0112-85	eBioscience	1/100
Anti-CD21 PE	eBio8D9	12-0211-82	eBioscience	1/200
Anti-PD-1 PE	J43	12-9985-82	eBioscience	1/200
Anti-CD4 PE	GK1.5	12-0041-82	eBioscience	1/400
Anti-CXCR4 PE	2B11	12-9991-82	eBioscience	1/200
Anti-CD40 PE	1C10	12-0401-82	eBioscience	1/200
Anti-CD69 PerCP-Cy5.5	H1.2F3	45-0691-82	eBioscience	1/200
Anti-Foxp3 PerCP-Cy5.5	FJK-16s	45-5773-82	eBioscience	1/100
Anti-Foxp3 APC	FJK-16s	17-5773-82	eBioscience	1/100
Anti-IFN-γ APC	XMG1.2	17-7311-82	eBioscience	1/200
Anti-Ki-67 eFlur660	SolA15	50-5699-82	eBioscience	1/600
Anti-GL7 eFluor660	GL7	50-5902-82	eBioscience	1/300
Anti-CD39 PerCP-eFluor710	24DMS1	46-0391-82	eBioscience	1/400
Anti-CTLA-4 PE	UC10-4B9	12-1522-82	eBioscience	1/75
Anti-CD49b PE	DX5	12-5971-82	eBioscience	1/200

Anti-MHC Class II I-Ab PerCP-eFluor710	AF6-120.1	46-5320-82	eBioscience	1/200
Anti-CD11c PE-Cy7	N418	25-0114-82	eBioscience	1/300
Anti-CD11c-FITC	HL-3	553801	BD Biosciences	1/100
Anti-CD45.1-APC-Cy7	A20	560579	BD Biosciences	1/200
Anti-CD45.2-PerCP-Cy5.5	104	552950	BD Biosciences	1/200
Anti-IL-17-PE	TC11-18H10	559502	BD Biosciences	1/200
Anti-Bcl-6-PE-Cy7	K112-91	563582	BD Biosciences	1/75
Anti-CD80-PE	16-10A1	553769	BD Biosciences	1/200
Anti-CD138-APC	281-2	558626	BD Biosciences	1/200
Anti-FAS (CD95)-PE	Jo2	554258	BD Biosciences	1/200
Anti-FAS (CD95)-PE-Cy7	Jo2	557653	BD Biosciences	1/200
Anti-FAS (CD95)-Biotin	Jo2	554256	BD Biosciences	1/200
Anti-CXCR5-Biotin	2G8	551960	BD Biosciences	1/75
Anti-IgD FITC	11-26c.2a	553439	BD Biosciences	1/100
Anti-CD38 PE	90	553764	BD Biosciences	1/200
Anti-Blimp-1-PE	C-21	sc-47732	Santa Cruz	1/75
Anti-CD4-Alexa Fluor 488	RM4-5	MCD0420	Invitrogen	1/200
Anti-CD3-Alexa Fluor 647	500A2	HM3421	Invitrogen	1/200
Anti-IgG(H+L)-Alexa Fluor 594	Polyconal	A-11007	Invitrogen	1/200
Streptavidin PerCP-Cy5.5	ND	405214	BioLegend	1/200
Streptavidin APC	ND	405207	BioLegend	1/400
Streptavidin PE	ND	554061	BD Biosciences	1/400
PNA-FITC	ND	FL-1071	Vector Laboratories	1/200
PNA-Alexa Fluor 647	ND	L32460	Invitrogen	1/400

Supplementary Table 1. Antibodies and staining reagents used in this study.
